# A Retrospective Study of the Safety and Immunogenicity of MVC-COV1901 Vaccine for People Living with HIV

**DOI:** 10.3390/vaccines11010018

**Published:** 2022-12-21

**Authors:** Shu-Hsing Cheng, Chia En Lien, Szu-Min Hsieh, Chien-Yu Cheng, Wang-Da Liu, Ching-Lung Lo, Wen-Chien Ko, Yen-Hsu Chen, Ching-Tai Huang, Hsiao-Ting Chang, Shinn-Jang Hwang, Ning-Chi Wang, Ming-Che Liu, Yu-Lin Lee, I-Chen Tai, Josue Antonio Garcia Estrada, Tzou-Yien Lin, Wen-Sen Lee

**Affiliations:** 1 Department of Infectious Diseases, Taoyuan General Hospital, Ministry of Health and Welfare, Taoyuan 330, Taiwan; 2 School of Public Health, Taipei Medical University, Taipei 110, Taiwan; 3 Medigen Vaccine Biologics, Taipei 114, Taiwan; 4 Institute of Public Health, College of Medicine, National Yang Ming Chiao Tung University, Taipei 112, Taiwan; 5 Department of Internal Medicine, Division of Infectious Diseases, National Taiwan University Hospital, Taipei 100, Taiwan; 6 College of Medicine, National Taiwan University, Taipei 106, Taiwan; 7 Department of Medicine, National Taiwan University Cancer Center, Taipei 100, Taiwan; 8 Department of Internal Medicine, National Cheng Kung University Hospital, Tainan 704, Taiwan; 9 Department of Medicine, College of Medicine, National Cheng Kung University, Tainan 701, Taiwan; 10 Department of Internal Medicine, Division of Infectious Diseases, Kaohsiung Medical University Hospital, Kaohsiung 807, Taiwan; 11 School of Medicine, Graduate Institute of Medicine, Sepsis Research Centre, Kaohsiung Medical University, Kaohsiung 807, Taiwan; 12 Centre of Tropical Medicine and Infectious Diseases, Kaohsiung Medical University, Kaohsiung 807, Taiwan; 13 Department of Infectious Diseases, Chang Gung Memorial Hospital, Taoyuan 333, Taiwan; 14 College of Medicine, Chang Gung University, Taoyuan 333, Taiwan; 15 Department of Family Medicine, Taipei Veterans General Hospital, Taipei 112, Taiwan; 16 School of Medicine, National Yang Ming Chiao Tung University, Taipei 112, Taiwan; 17 Tri-Service General Hospital, Taipei 114, Taiwan; 18 Clinical Research Centre, Taipei Medical University Hospital, Taipei 110, Taiwan; 19 School of Dental Technology, College of Oral Medicine, Taipei Medical University, Taipei 110, Taiwan; 20 Department of Internal Medicine, Changhua Christian Hospital, Changhua 500, Taiwan; 21 Program in Medical Biotechnology, National Chung Hsing University, Taichung 402, Taiwan; 22 Department of Paediatrics, Chang Gung Memorial Hospital, Taoyuan 333, Taiwan; 23 Division of Infectious Disease, Department of Internal Medicine, Wan Fang Hospital, Taipei Medical University, Taipei 116, Taiwan; 24 Department of Internal Medicine, School of Medicine, College of Medicine, Taipei Medical University, Taipei 110, Taiwan

**Keywords:** COVID-19 vaccine, CpG 1018, S-2P protein, HIV, CD4/CD8 ratio, immunogenicity

## Abstract

Background: This study aimed to assess the safety and immunogenicity of MVC-COV1901, a recombinant COVID-19 protein vaccine, containing S-2P protein adjuvanted with CpG 1018 and aluminum hydroxide, for people living with HIV (PWH). Methods: A total of 57 PWH of ≥20 years of age who are on stable antiretroviral therapy were compared with 882 HIV-negative participants. Participants received two doses of MVC-COV1901 28 days apart. Results: No vaccine-related serious adverse events (SAEs) were recorded. Seroconversion rates (SCRs) of 100% and 99.8% were achieved in PWH and comparators, respectively, 28 days after the second dose. After adjusting for sex, age, BMI category, and comorbidity, the adjusted GMT ratio of comparator/PWH was 3.2 (95% CI 2.5–4). A higher CD4/CD8 ratio was associated with a higher GMT (R = 0.27, *p* = 0.039). MVC-COV1901 has shown robust safety but elicited weaker immune responses in PWH. Conclusions: Further investigations may be needed to determine whether PWH require distinct immunization strategies with improved immunogenicity. The main study is registered at ClinicalTrials.gov (NCT04695652).

## 1. Introduction

The Coronavirus Disease 2019 (COVID-19), caused by the novel coronavirus SARS-CoV-2, was first reported in Wuhan, Hubei Province, People’s Republic of China (PRC) in late December 2019. The disease spread rapidly around the globe and was declared a pandemic by the WHO on 11 March 2020 [1,2]. As of November 2022, nearly 643 million infections and over 6 million deaths have occurred worldwide [3]. Unfortunately, this disease has severely impacted people with poor socioeconomic status, and that includes populations vulnerable to HIV infection [3,4].

People who are living with HIV (PWH) are subject to many risk factors that predispose them to severe COVID-19. These factors include high rates of smoking, drug use, comorbidities including cardiovascular diseases, diabetes mellitus, chronic renal diseases, chronic liver diseases, and lung diseases [5,6,7,8]. Furthermore, many PWH are overweight or obese, which correlates with the widespread use of second-generation integrase inhibitors [9,10] and is an additional risk factor for severe COVID-19 [11,12]. Health disparities due to socioeconomic status and stigmatization may also hinder the early diagnosis of SARS-CoV-2 infection and the timely provision of healthcare [4]. Several case series and meta-analyses also pointed towards the association between PWH and severe COVID-19 and higher mortality rates. The U.S. RedCap data showed that COVID-19 affects PWH that are predominantly of older age (mean 51.4 years), African-American (47.5%), and having high rates of comorbidity (80%), with 57.3% greater risk for hospitalization, 16.5% for ICU admission, and 9.4% for mortality [13]. Data captured from the ISARIC WHO CCP study showed that after adjusting for age, PWH have 47% higher mortality rates (adjusted hazard ratio 1.47, and 95% confidence interval [CI] 1.04–2.25) in England [14]. The astonishingly rapid spread of COVID-19 and expansion of several variants of concern (VOCs), such as Alpha, Beta, Delta, Gamma, and Omicron variants, spurred the desperate need of creating an effective COVID-19 vaccination strategy for PWH.

MVC-COV1901 is a subunit vaccine based on the stabilized prefusion SARS-CoV-2 spike protein S-2P (15 mcg) with furin cleavage site mutation and T4 fibritin for trimerization, and it formulated in adjuvant composed of a toll-like receptor 9 (TLR9) agonist CpG 1018 and aluminium hydroxide. It is based on the ancestral strain of the SARS-CoV-2 virus [15,16,17]. Previous phase 1 and 2 clinical trials have shown that the MVC-COV1901 vaccine was well-tolerated and elicited robust immune responses [18,19]. In this study, PWH, matched with HIV-negative subjects, were inoculated with MVC-COV1901 and compared in terms of safety and immunogenicity.

## 2. Materials and Methods

### 2.1. Study Design and Participants

**Figure 1:**
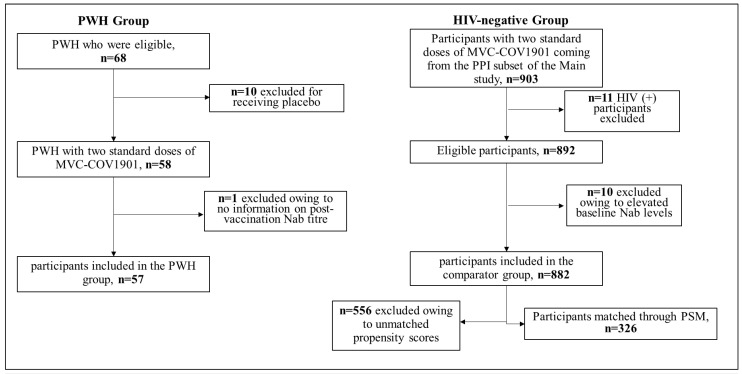
The algorithm of the PWH group and the HIV-negative group receiving the MVC-COV1901 protein vaccine. Abbreviations: PWH = People Living with HIV; Nab = neutralizing antibodies; HIV = Human Immunodeficiency Virus; PSM = Propensity Score Matching.

This is a substudy within a Phase 2, prospective, double-blinded, and multi-centre study to evaluate the SARS-CoV-2 vaccine MVC-COV1901. During enrolment, individuals underwent clinical laboratory tests and physical examinations including evaluation of medical history. Existing HIV infection was noted. The main Phase 2 study started on 30 December 2020, for which the interim report was completed in June 2021 [19]. Retrospective data for PWH of the substudy was collected between 24 September and 15 November 2021. The PWH included were on stable antiretroviral therapy with CD4+ T cell count greater than 350 cells/mm^3^ and HIV viral load less than 103 copies/mL. We compared PWH from a per-protocol immunogenicity (PPI) subset of the main study with HIV-negative participants of the main study (Figure 1). There were 57 individuals belonging to the HIV-positive group while 882 were included in the HIV-negative group. A total of 326 HIV-negative individuals were included in the comparator post-propensity score matching. Participants’ ages ranged from 20–87, and all received two standard doses of 15 mcg MVC-COV1901, administered 28 days apart via IM injection.

Immediate adverse events (AEs), both solicited local and systemic, unsolicited AEs, and adverse events of special interest (AESIs) were recorded throughout the study period. For the safety data, the full safety set of participants from the main study was included in the analysis and compared to the HIV-positive group. The safety set involved participants in the main study who had at least one dose of the MVC-COV1901. A comprehensive evaluation of the safety profile of the two groups includes the recording of adverse events of people who received at least one dose of the study intervention, thus adverse events that occurred in the HIV-positive group were compared to the adverse events that occurred in the full safety set of the main study. For the safety profile comparison, we wanted to have as much data as possible to detect any unusual safety signals. Since in the process of propensity score matching, the unmatched individuals were discarded, to avoid the full safety picture being compromised, we chose to show the full scope of comparison.

Immunogenicity was assessed by measuring GMTs and seroconversion rates (SCRs) of neutralizing antibody. The detection and characterization of neutralizing antibodies were performed with central laboratories using validated pseudovirus and/or live virus neutralization assays [19]. To measure neutralizing antibody titers, wildtype SARS-CoV-2, Taiwan CDC strain number 4 (hCoV-19/Taiwan/4/2020; Global Initiative on Sharing All Influenza Data accession ID EPI_ISL_411927), was titrated to calculate the 50% tissue culture infective dose (TCID_50_). The hCoV-19/Taiwan/4/2020 is identical to the prototype virus strain. Vero E6 cells were seeded in 96-well plates (at 1.2 × 10⁴ cells per well) and incubated. Starting from a 1:8 dilution, the serum samples were diluted two-fold eight times to a final dilution of 1:1024. The diluted serum samples were then mixed with an equal volume of 100 TCID_50_ per 50 μL of virus. After incubating the serum–virus mixture at 37 °C for 1 h, it was added to the wells containing Vero E6 cells. The cells were then incubated at 37 °C in a 5% CO_2_ incubator for 4–5 days. The neutralizing titer (NT_50_) was defined as the reciprocal of the highest dilution capable of inhibiting 50% of the cytopathic effect. The NT_50_ results were derived from quadruplicates and calculated with the Reed-Muench method. Neutralizing antibody titers were converted to the WHO Standardized Unit, IU/mL. The conversion is based on the WHO validated NIBSC reference panel. The conversion formula was established based on the NT_50_ data of measuring the standards with defined IU/mL, including WHO SARS-CoV-2 international standard 20/136, WHO reference panel 20/268, and also the first international standard 20/130 before WHO IS 20/136 was launched. Each of the standards was measured 3 times (as 3 independent samples).

Written informed consent was obtained from all participants. The trial protocol and informed consent form were approved by the Taiwan Food and Drug Administration (TFDA) and the ethics committees at the conducting sites: National Taiwan University Hospital, Tri-Service General Hospital, Taipei Veterans General Hospital, Taipei Medical University Hospital, Taipei Municipal Wan Fang Hospital, Linkou Chang Gung Memorial Hospital, Taoyuan General Hospital Ministry of Health and Welfare, China Medical University Hospital, Changhua Christian Hospital, National Cheng Kung University Hospital, and Kaohsiung Medical University Chung-Ho Memorial Hospital. This trial was conducted in accordance with the principles of the Declaration of Helsinki and Good Clinical Practice (GCP) guidelines.

### 2.2. Statistical Analysis

For the statistical analyses, descriptive statistics were first obtained and used to present sociodemographic and other characteristics. SCRs and 95% confidence interval (CI) were computed for individuals with at least a fourfold increase from the baseline. Fisher’s exact test was used to compare seroconversion between the HIV group and the main group. GMTs were estimated from neutralizing antibody titers measured at 28 days after the second dose of the study intervention. GMT ratios, computed as the ratio between the GMT of neutralizing antibodies in the HIV subgroup versus the control group, were also estimated. To assess the magnitude of the difference in immune response between the two groups, an analysis of the covariance (ANCOVA) model was used. The model included the log-transformed antibody titers at Day 57 as the dependent variable and the group (HIV subgroup and main group) as an explanatory variable, and they were adjusted for age, BMI, gender, and comorbidity profile. The 95% CI for the adjusted neutralizing antibody titers of each vaccine group was obtained. Then, the adjusted GMT was back-transformed to the original scale. The adjusted GMT ratio of the two groups and the corresponding 95% CI were also estimated. The correlation between GMT and CD4/CD8 ratio, and GMT and absolute CD4+ T cell count were analyzed using Spearman’s test. A one-way ANOVA was applied using log-transformed Nab titers for calculating the association between GMT and HIV classification stages. Out of the 57 HIV-positive individuals, three were excluded from this analysis due to a lack of information on the HIV classification stage. Lastly, using age and gender as covariates and a digit-based greedy and nearest neighbor approach with a 1:5 ratio, propensity score matching was employed for robust comparison between the two groups. Propensity scores were first estimated based on a multivariable logit regression. Propensity scores for the HIV-positive (case) group were then matched to that of the HIV-negative (control) individuals in a ratio of 1:5 without replacement. Matching without replacement meant that a patient from the control group who was already matched to a patient from the case group was not eligible for matching to another individual from the case group. Only subjects with a propensity score within 25% of the standard deviation of a case’s propensity score were matched. Any unmatched control was discarded. Differences between treatment groups for each covariate were assessed before and after matching to determine if there was sufficient balance. Standardized mean differences were evaluated to determine imbalances. The demographic characteristics of the excluded individuals are presented in Table S1.

## 3. Results

### 3.1. Study Population

In the main study, a total of 3854 participants were randomized to treatment groups. Among them, 58 PWH were randomized to the MVC-COV1901 group (Figure 1). One PWH was excluded from the analytic sample due to a lack of information on an outcome indicator (i.e., neutralizing antibody titer). There were 882 participants who were HIV-negative and aged 20 to 87 in the PPI subset from the main study (Table 1). Propensity matching resulted in a total of 57 participants in the HIV-positive group and 326 individuals in the HIV-negative group (Figure 1).

The demographic characteristics of the 57 PWH and the 326 matched comparators from the main study are summarized in Table 1. The mean age of the HIV group and the main group was 38.6 (Interquartile range [IQR] 19.0) and 42.8 (IQR 23.0) years, respectively (*p* = 0.058); 94.7% and 95.1% were male, respectively (*p* = 1); 19.3% and 15.0% had BMI ≥ 30 kg/m^2^, respectively (*p* = 0.413); and 10.5% and 18.1% had comorbidities at baseline, respectively (*p* = 0.16) (Table 1).

### 3.2. Safety

Overall, the percentages of participants in each category (solicited adverse events, unsolicited adverse events, and other adverse events after vaccination) were comparable between the HIV group and the main group in all age groups (Table 2a,b). The percentages of participants that reported solicited local adverse events were 58.6% and 72.3% for the PWH and main group, respectively. For solicited systemic adverse events, 63.8% and 53.8% were reported for the PWH and main group, respectively. In both PWH and HIV-negative participants, pain or tenderness at the injection site was the most common reported local reaction (67.2% and 71.2%, respectively). This event was slightly less common among PWH than HIV-negative individuals. Among systemic reactions, malaise and headache were the most common reaction in both groups but were predominantly mild to moderate in intensity.

### 3.3. Immunogenicity

An assessment of immunogenicity among matched samples suggests that after two doses of study intervention, the wild-type SARS-CoV-2 neutralizing GMT’s ratio (95% CI) of HIV-negative/HIV-positive was 3.0 (95% CI 2.4–3.8) on Day 57 (i.e., 28 days after the 2nd dose), with 412.0 IU/mL (95% CI 378.7–448.3) in the HIV-negative group and 137.7 IU/mL (95% CI 110.7–171.3) in the HIV group, respectively (Table 3). After adjusting for sex, age, BMI category, and comorbidity profile, the adjusted GMT ratio (95% CI) of HIV-negative/HIV-positive was 3.2 (95% CI 2.5–4.0).

The seroconversion rates on Day 57 based on the wild-type SARS-CoV-2 GMT were 100% in the PWH group and 99.8% in the main group with only two participants in the group failing to seroconvert.

### 3.4. Correlation of GMT and CD4/CD8 Ratio

**Figure 2:**
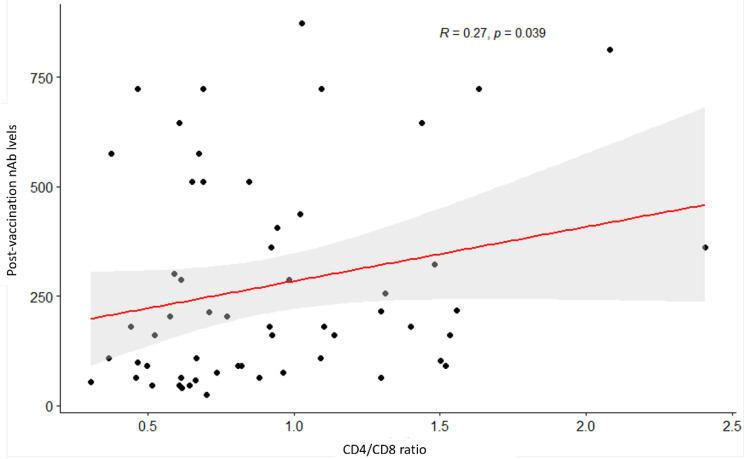
Scatterplot between CD4/CD8 ratio and post-vaccination neutralization antibody titers. *p* value (i.e., 0.039) for the Spearman’s test indicates a significant relationship at a 5% significance level.

**Figure 3:**
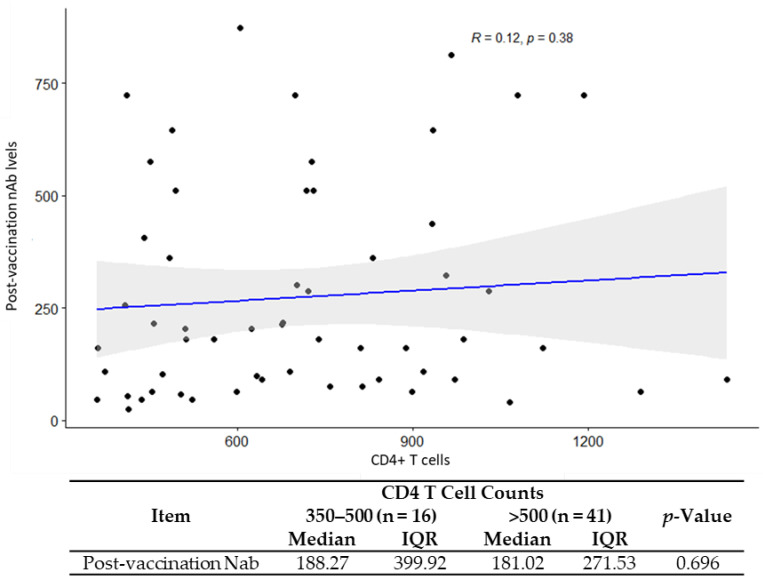
Correlation analysis for CD4+ T cell counts with post vaccination neutralization antibody titers.

CD4/CD8 ratio was positively associated with GMT (R = 0.27, *p* = 0.039) (Figure 2). No correlations were found between the GMTs and CD4+ T cell count (*p* = 0.3) and the GMT and HIV classification stages (*p* = 0. 35) (Figure 3 and Table 4).

## 4. Discussion

In this study, the safety and immunogenicity of recombinant S-2P protein vaccine against COVID-19 were assessed in PWH. The interim analysis demonstrated that in PWH participants aged 20 years and older, two doses of the MVC-COV1901 vaccine were safe and well-tolerated but less immunogenic than in HIV-negative controls. By the time the report was written, all participants had been followed up with for up to six months after the second booster dose.

Based on the resulting GMTs, the immunogenicity of MVC-COV1901 may be compared to that of two doses of AZD1222 among healthy individuals. In an immunobridging study, the GMT ratio of Nab’s between MVC-COV1901 and AZD1222 was 3.8 times with a 95% confidence interval of 3.4–4.3 [20]. This is similar to the adjusted Nab GMT ratio between HIV-negative and HIV-positive individuals inoculated with MVC-COV1901 (i.e., 3.2 [95% CI: 2.5–4.0]). Although no correlates of protection have been established yet, neutralizing antibody levels might indicate similar efficacy to AZD1222 [21]. Previous research raised the possibility that the immune status of PWH negatively modulates the immune responses to COVID-19 vaccines. Specifically, PWH have diminished or less durable responses to hepatitis B and yellow fever vaccination [22,23,24], and people with low CD4 cell counts have diminished antibody titers to pneumococcus, influenza, diphtheria, tetanus, and poliomyelitis [25,26,27]. Despite these observations, most trials for COVID-19 vaccines have not addressed the PWH subpopulation with a subgroup analysis or comparison of PWH with HIV-negative control groups [28,29,30,31,32]. Several studies exhibited the same strength of immune response and safety profile, refs. [33,34,35,36] compared to HIV-negative comparators; the others showed weaker immune responses [37,38,39,40]. (Table 4). Despite the abovementioned studies, the numbers of PWH participating in clinical trials that evaluated COVID-19 vaccines is still very limited [41]. Weaker neutralization antibody responses to the Spike protein were demonstrated in this study, similar to the protein-based vaccine, NVX-CoV2373, studied in South Africa, ref. [38] and the inactivated SARS-CoV-2 vaccine studied in China [39].

CD4+ T cells orchestrate the response to acute and chronic viral infections by coordinating the immune system. These cells activate B cells to generate the efficient neutralisation antibodies, cytotoxic CD8+ T cells to kill infected cells, and multiple cells of the innate immune system and non-immune cells. Thus, CD4+ T cells play a key role for the establishment of long-term cellular and humoral antigen-specific immunity, which is the basis of life-long protection for many viral infections and vaccines [42,43]. In addition, CD4+ and CD8+ T cells produce interferon-gamma (commonly referred to as a “type 1” immune response), which is believed to be protective for the host [44]. It is therefore a legitimate concern that the immune response could be impeded in PWH with abnormal T cell counts as measured by depleted memory T cells and inversed CD4+/CD8+ ratios that may be indicative of the response of exhausted cytotoxic T cells toward HIV and persistent immune activation and inflammation even during stable antiretroviral therapy (ART) [45,46,47]. Nevertheless, the generation of neutralizing antibodies was a key endpoint in this vaccine study.

Notably, in the study with MVC-COV1901 presented here, CD4+ T cell counts did not significantly correlate with GMT in the vaccinated PWH while increasing CD4/CD8 ratios did correlate, unlike other studies [37,40]. Despite this, however, a “weaker” correlation was seen between GMT and CD4/CD8 ratios, which may partly be explained by the small size of the HIV-positive group and other factors of HIV disease not taken into account. Nevertheless, the adjuvant of MCV-COV1901, CpG 1018 (a toll-like receptor 9 agonist), may explain this correlation because it binds to the DNA receptor on plasmacytoid dendritic cells and enhances immunogenicity by stimulating CD4+ helper and CD8+ cytotoxic T cells simultaneously [48,49]. Consistent with these observations, an independent HBV vaccine study in PWH demonstrated that CD4/CD8 ratios > 0.4 were associated with a high rate (86%) of HBV seroconversion [50].

Despite these indications that cellular immunity may be important for durable protective immunity, there are arguments that long-lived plasma cells may have the potential to produce antibodies for decades in the absence of a re-encounter with the antigen or specific T cells [51]. Add to this the 100% of seroconversion in PWH with MVC-COV1901, and it is clear that the role of T cell memory in durable protective immunity against SARS-CoV-2 deserves further study.

## 5. Limitation

Despite the insights generated by our study, some limitations to interpretation may exist. First, the sample size in the PWH group was relatively small. Furthermore, all PWH were on stable ART and had CD4+ T cell counts greater than 350 cells/mm^3^ and HIV viral load less than 1000 genome copies/mL. Thus, extrapolation to people with HIV with lower CD4 counts or without suppressed HIV viral loads is not suggested. Recent studies have reported PWH with CD4+ T cell counts less than 200 cells/mm^3^ presented weaker immunologic responses [37,38,39,40,41]. Second, our study was initiated when SARS-CoV-2 was not endemic in Taiwan, and the low viral transmission rate made it difficult to ascertain the efficacy of the vaccine as an exploratory endpoint. Specifically, low levels of 1% of neutralizing antibody titer were detected both at baseline and on Day 57 in the placebo group, suggesting that COVID-19 was rare, and natural infection had not boosted the neutralizing antibody titers [19]. Third, the short duration of the follow-up period in this study did not allow for assessing the durability of immune responses after Day 57. Fourth, although Th1-skewed immune responses had been demonstrated in the phase I MVC-COV1901 study 18, the T-cell responses to the vaccine among PWH were not assessed in this study. Finally, neutralization activities for emerging VOCs were not tested, and the cross-reactivity remains unknown.

## 6. Conclusions

This report describes a good safety profile but weaker immunogenicity of MCV-COV1901 in PWH, especially in those PWH with low CD4/CD8 ratios. MCV-COV1901 has emergency authorization use in Taiwan as of 19 July 2021 and has since advanced to larger clinical trials, including a trial initiated by the WHO [52]. Additional information accumulates from these trials, but further studies are needed to see if PWH require distinct immunization strategies with improved immunogenicity, such as boosters or additional doses [53,54], heterologous revaccination [55], or higher doses as with hepatitis B [56] and that for the influenza vaccine [57]. These studies may include, at this point, effectiveness studies focusing on PWH, which may justify the use of strategies such as additional doses of the vaccine or heterologous boosting. Future research should also investigate the effectiveness of vaccines among PWH who have higher levels of immunodeficiency and PWH against Omicron or future variants [58].

## Figures and Tables

**Table 1:** Demographic characteristics of the matched participants.

Item	HIV Positive N = 57	Main Study N = 326	*p*-Value
Age (years)			
Mean (SD)	38.6 (13.1)	42.8 (14.9)	0.058
Median (IQR)	36 (19.0)	41 (23.0)	
Min~Max	23.0~72.0	23.0~72.0	
Gender			
N (Missing)	57 (0)	326 (0)	
Male	54 (94.7%)	310 (95.1%)	1
Female	3 (5.3%)	16 (4.9%)	
BMI (kg/m^2^)			
Mean (SD)	25.3 (4.9)	25.9(4.0)	0.167
Median (IQR)	24.5 (6.2)	25.4 (5.1)	
BMI group			
<30 kg/m^2^	46 (80.7%)	277 (84.9%)	0.413
≥30 kg/m^2^	11 (19.3%)	49 (15.0%)	
Comorbidity Category			
Yes	6 (10.5%)	59 (18.1%)	0.16
No	51 (89.5%)	267 (81.9%)	

Abbreviations: N = number of subjects in PPI population; SD = standard deviation; Q1 = first quartile (25th percentile); Q3 = third quartile (75th percentile); IQR = interquartile range; BMI = Body Mass Index; HIV = Human Immunodeficiency Virus.

**Table 2:** (**a**) Solicited adverse events after any dosing. (**b**) Summary of unsolicited adverse events and other adverse events.

**(a)**						
	**All, n (%)**		**≥20 to <65 Years, n (%)**		**≥65 Years, n (%)**	
	**MVC-COV1901** **(N = 3295)**	**HIV** **(N = 58)**	**MVC-COV1901** **(N = 2575)**	**HIV** **(N = 54)**	**MVC-COV1901** **(N = 720)**	**HIV** **(N = 4)**
Any Solicited Local AEs	2381 (72.3)	39 (58.6)	2030 (78.8)	37 (59.3)	351 (48.8)	2 (50.0)
Pain/Tenderness	2346 (71.2)	39 (67.2)	2009 (78.0)	37 (68.5)	337 (46.8)	2 (50.0)
Grade 1	2237 (67.9)	37 (63.8)	1907 (74.1)	35 (64.8)	330 (45.8)	2 (50.0)
Grade 2	97 (2.9)	2 (34.4)	91 (3.5)	2 (3.7)	6 (0.8)	0
Grade 3	12 (0.4)	0	11 (0.4)	0	1 (0.1)	0
Induration/Swelling	347 (10.5)	1 (1.7)	286 (11.1)	1 (1.9)	61 (8.5)	0
Grade 1	303 (9.2)	1 (1.7)	246 (9.6)	1 (1.9)	57 (7.9)	0
Grade 2	42 (1.3)	0	38 (1.5)	0	4 (0.6)	0
Grade 3	2 (0.1)	0	2 (0.1)	0	0	0
Erythema/Redness	161 (4.9)	0	138 (5.4)	0	23 (3.2)	0
Grade 1	155 (4.7)	0	132 (5.1)	0	23 (3.2)	0
Grade 2	6 (0.2)	0	6 (0.2)	0	0	0
Any Solicited Systemic AEs	1774 (53.8)	37 (63.8)	1484 (57.6)	35 (65.8)	290 (40.3)	2 (50.0)
Malaise/Fatigue	1186 (36.0)	25 (43.1)	1036 (40.2)	24 (44.4)	150 (20.8)	1 (25.0)
Grade 1	961 (29.2)	22 (43.1)	831 (32.3)	24 (44.4)	130 (18.1)	1 (25.0)
Grade 2	203 (6.2)	3 (5.2)	185 (7.2)	2 (3.7)	18 (2.5)	1 (25.0)
1Grade 3	22 (0.7)	0	20 (0.8)	0	2 (0.3)	0
Myalgia	908 (27.6)	10 (16.7)	757 (29.4)	14 (25.9)	151 (21.0)	2 (50.0)
Grade 1	764 (23.2)	9 (16.7)	629 (24.4)	7 (13.0)	135 (18.8)	2 (50.0)
Grade 2	126 (3.8)	1 (1.7)	112 (4.3)	1 (1.9)	14 (1.9)	0
Grade 3	18 (0.5)	0	16 (0.6)	0	2 (0.3)	0
Headache	730 (22.2)	13 (22.4)	631 (24.5)	12 (22.2)	99 (13.8)	1 (25.0)
Grade 1	630 (19.1)	12 (20.7)	537 (20.9)	11 (20.4)	93 (12.9)	1 (25.0)
Grade 2	93 (2.8)	1 (1.7)	87 (3.4)	1 (1.9)	6 (0.8)	0
Grade 3	7 (0.2)	0	7 (0.3)	0	0	0
Diarrhoea	497 (15.1)	14 (24.1)	422 (16.4)	14 (25.9)	75 (10.4)	0
Grade 1	411 (12.5)	12 (20.7)	350 (13.6)	12 (22.2)	61 (8.5)	0
Grade 2	75 (2.3)	2 (3.4)	62 (2.4)	2 (3.7)	13 (1.8)	0
Grade 3	11 (0.3)	0	10 (0.4)	0	1 (0.1)	0
Nausea/Vomiting	254 (7.7)	6 (10.3)	219 (8.5)	6 (11.1)	35 (4.9)	0
Grade 1	226 (6.9)	6 (10.3)	192 (7.5)	6 (11.1)	34 (4.7)	0
Grade 2	26 (0.8)	0	25 (1.0)	0	1 (0.1)	0
Grade 3	2 (0.1)	0	2 (0.1)	0	0	0
Fever	23 (0.7)	0	16 (0.6)	0	7 (1.0)	0
Grade 1	14 (0.4)	0	8 (0.3)	0	6 (0.8)	0
Grade 2	6 (0.2)	0	6 (0.2)	0	0	0
Grade 3	2 (0.1)	0	2 (0.1)	0	0	0
Grade 4	1 (<0.1)	0	0	0	1 (<0.1)	0
**(b)**						
	**All, n (%)**		**≥** **20 to <65 Years, n (%)**		**≥65 Years, n (%)**	
	**MVC-COV1901** **(N = 3295)**	**HIV in MVC-COV1901** **(N = 58)**	**MVC-COV1901** **(N = 2575)**	**HIV in MVC-COV1901** **(N = 54)**	**MVC-COV1901** **(N = 720)**	**HIV in MVC-COV1901** **(N = 4)**
Unsolicited AEs	932 (28.3)	17 (29.3)	767 (29.8)	17 (29.6)	165 (22.9)	0
Related unsolicited AEs	406 (12.3)	8 (13.8)	340 (13.2)	396 (13.2)	66 (9.2)	0
Unsolicited AEs ≥ Grade 3	93 (2.8)	3 (5.2)	86 (3.3)	96 (3.2)	7 (1.0)	0
Related unsolicited AEs ≥ Grade 3	21 (0.6)	0	20 (0.8)	24 (0.8)	1 (0.1)	0
SAEs	18 (0.5)	1 (1.7)	16 (0.6)	17 (0.6)	2 (0.3)	0
Related SAEs	0	0	0	0	0	0
AESI	1 (<0.1)	0	0	0	1 (0.1)	0
VAED	0	0	0	0	0	0
AEs leading to study intervention discontinuation	2 (0.1)	0	0	1 (<0.1)	2 (0.3)	0
AEs leading to study withdrawal	1 (<0.1)	0	0	0	1 (0.1)	0
Death	0	0	0	0	0	0

Abbreviations: AE = adverse event; AESI = adverse events of special interest; CI = confidence interval; N = number of participants in the population; n = number of participants with events; SAE = serious adverse event; VAED = vaccine-associated enhanced disease.

**Table 3:** Comparison of wild-type SARS-CoV-2 neutralizing antibody (Nab) in IU/mL among matched participants.

Item	Nab Titer (GMT), IU/mL		^a^ Nab GMT Ratio	^a,b^ Adjusted GMT Ratio
	HIV-Positive (n = 57)	HIV-Negative (n = 326)		
Estimate	137.7	412.0	3.0	3.2
95% CI	110.7~171.3	378.7~448.3	2.4~3.8	2.5~4.0

^a^ GMT_HIV-negative_/GMT_HIV-positive_ ratio; ^b^ Estimated using ANCOVA model where sex, age, BMI category, and comorbidity profile are covariates; Abbreviations: HIV = Human Immunodeficiency Virus; Nab = Neutralizing antibody; GMT = Geometric mean titer.

**Table 4:** Correlation analysis for HIV classification stage with post vaccination neutralization antibody titers.

HIV Classification Stage	n	Nab GMT (in IU/mL)	95% CI	* *p*-Value
Stage 1	25	142.55	105.34–192.90	0.3486
Stage 2	19	108.54	71.00–165.94	
Stage 3	10	169.05	85.40–334.65	

* using one-way ANOVA; Abbreviations: HIV = Human Immunodeficiency Virus, n = number of participants, Nab = neutralizing antibody, GMT = Geometric mean titers, CI = Confidence interval.

## Data Availability

The datasets generated and/or analyzed during the current study are not publicly available as it is an interim analysis of data from an ongoing study.

## Supplementary Materials

The following supporting information can be downloaded at: https://www.mdpi.com/article/10.3390/vaccines11010018/s1, Figure S1: Propensity scores for people living with HIV, comparing unmatched participants from the main study, and comparing controls with matched propensity scores; Table S1: Demographic characteristics for the group from the main study and controls with matched propensity scores.
