# Supplementary material for: A Retrospective Study of the Safety and Immunogenicity of MVC-COV1901 Vaccine for People Living with HIV
Source: Vaccines (Basel). 2022 Dec 21;11(1):18. doi: 10.3390/vaccines11010018 (PMC9863561; doi:10.3390/vaccines11010018)
Supplement: Supplementary file 1 [file vaccines-11-00018-s001.zip › vaccines-2064078-supplementary.pdf]

Supplementary material

1. Figure S1. Propensity scores for people living with HIV, comparing unmatched participants from the main study, and comparing controls with matched propensity scores.
2. Table S1. Demographic characteristics for the group from the main study and controls with matched propensity scores.

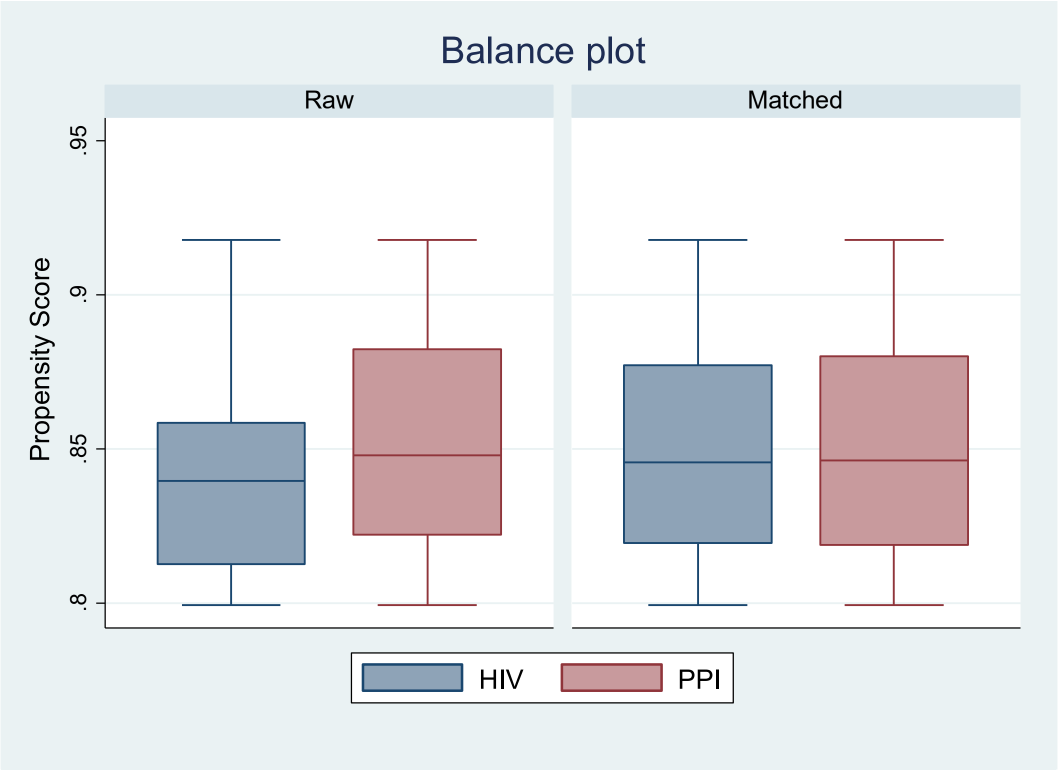

Figure S1. Propensity scores for people living with HIV, comparing unmatched participants from the main study, and comparing controls with matched propensity scores.

Table S1. Demographic characteristics for the group from the main study and controls with matched propensity scores.

Item	MVC-COV1901<br>Main study total PPI sample	MVC-COV1901<br>Matched sample
**·Age (years)**		
N (Missing)	556 (0)	326 (0)
Mean (SD)	47.4 (17.4)	42.8 (14.9)
Median (IQR)	45 (34.0)	41 (23.0)
Q1~Q3	31.0-65.0	30.0~53.0
Min~Max	20.0~87.0	23.0~72.0
**·Gender**		
N (Missing)	57 (0)	326 (0)
Male	361(64.9%)	310 (95.1%)
Female	195 (35.1%)	16 (4.9%)
**·BMI (kg/m^2)**		
N (Missing)	556 (0)	326(0)
Mean (SD)	24.3(4.0)	25.9(4.02)
Median (IQR)	23.3(5.2)	25.4 (5.12)
Q1~Q3	21.5~26.7	23.2~28.3
Min~Max	14.4~45.2	16.6~40.5
**·BMI group**		
N (Missing)	556 (0)	326 (0)
<30 kg/m^2	504 (90.7%)	277 (84.97)
>=30 kg/m^2	52 (9.35%)	49 (15.03)
**·Comorbidity Category**		
N (Missing)	556 (0)	326(0)
Yes	112 (20.14%)	59 (18.1%)
No	112 (20.1%)	267 (81.9%)

(1) Abbreviations: N=number of subjects in PPI population; SD=standard deviation; Q1=first quartile (25th percentile); Q3=third quartile (75th percentile); IQR=interquartile range; BMI=Body Mass Index; HIV=Human Immunodeficiency Virus.
